# Endocardite infectieuse sur communication interventriculaire: intérêt de l’échocardiographie? de l’antibioprophylaxie?

**DOI:** 10.11604/pamj.2016.25.154.10673

**Published:** 2016-11-14

**Authors:** Hanane Boussir, Amine Ghalem, Nabila Ismaili, Noha El Ouafi

**Affiliations:** 1 Service de Cardiologie, Centre Hospitalier universitaire Mohammed VI, Oujda, Maroc

**Keywords:** CIV, endocardite infectieuse, prophylaxie des EI, recommandations ESC 2015, VSD, infective endocarditis, IE prophylaxis, 2015 ESC Guidelines

## Abstract

L’endocardite infectieuse peut survenir sur un cœur sain ou pathologique. Parmi les cardiopathies à risque, on trouve les cardiopathies congénitales dont la CIV est la plus fréquente. Nous rapportons le cas d’une endocardite infectieuse à streptocoque oral, survenue sur une CIV non connue jusqu’à maintenant, chez un patient de 17 ans, se présentant sous forme de fièvre prolongée associée à une éruption cutanée. L’examen de la sphère ORL révéla par ailleurs des angines pseudomembraneuses avec un mauvais état buccodentaire. Les EI sur CIV sont les plus fréquentes des EI sur cardiopathie congénitale. Leur présentation clinique peut être atypique d’où le rôle primordial de l’échocardiographie. La prévention dans ces cas passe par une hygiène bucco-dentaire et cutanée optimale et non par une antibioprophylaxie.

## Abstract (en)

Infective endocarditis can occur in healthy people or in those with a preexisting heart disease. Among people with heart disease at risk for developing infective endocarditis, there are those with congenital heart disease (VSD is the most common type). We report the case of a 17-year old patient with infective endocarditis due to oral streptococci associated with VSD that wasn’t detected until then, whose symptoms were prolonged fever accompanied by a rash. ENT examination also showed pseudomembranous anginas associated with poor oral health status. IE associated with VSD are the most frequent IEs among patients with congenital heart disease. Their clinical presentation can be atypical, hence the vital role of echocardiography. In these cases effective prevention requires optimal oral and skin hygiene and not prophylactic antibiotic therapy.

## Introduction

L’endocardite infectieuse (EI) est une atteinte infectieuse de l’endocarde causant des dégâts essentiellement valvulaires, responsable d’une mortalité et d’une morbidité importante. Elle peut survenir sur un cœur sain ou pathologique. Parmi les cardiopathies à risque, on trouve les cardiopathies congénitales dont la CIV est la plus fréquente.

## Patient et observation

**Figure 1:**
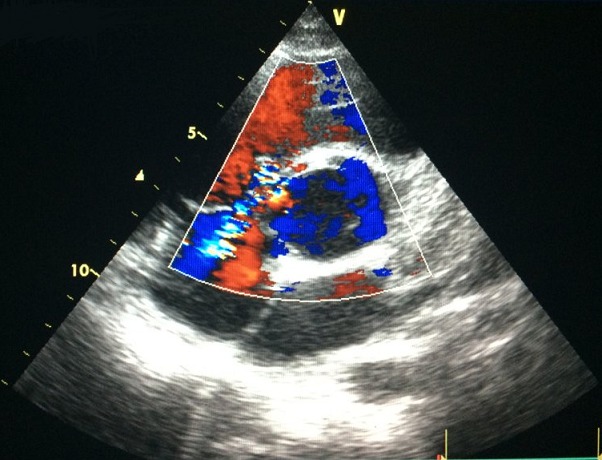
Incidence parasternale petit axe montrant un shunt gauche-droit interventriculaire au doppler couleur

**Figure 2:**
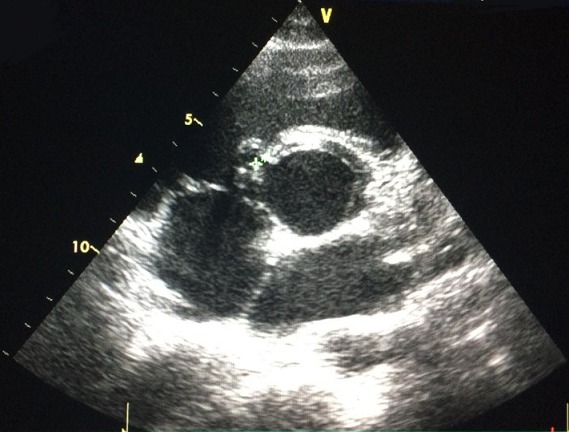
Incidence parasternale petit axe montrant une CIV périmembraneuse de 3 mm

**Figure 3:**
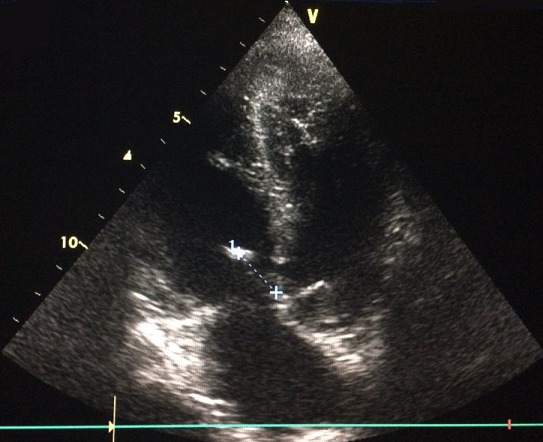
Incidence apicale 4 cavités montrant une grosse végétation de 17mm insérée sur la berge supérieure de la CIV

**Figure 4:**
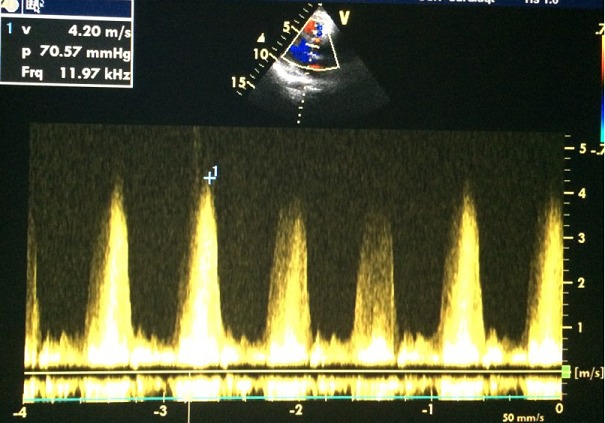
Flux restrictif de la CIV au Doppler continu

Il s’agit d’un patient de 17 ans admis pour prise en charge d’une fièvre prolongée associée à une éruption cutanée. Nous avons relevé comme antécédent une allergie à la pénicilline découverte à l’âge de 11 ans. L’examen générale à l’admission trouvait un patient fébrile à 38,5°C, un exanthème généralisé, un purpura vasculaire au niveau des deux membres inférieurs. L’auscultation cardiaque trouvait un souffle systolique en rayon de roue. L’examen de la sphère ORL révéla par ailleurs des angines pseudomembraneuses avec un mauvais état buccodentaire. Au bilan biologique, on trouvait un franc syndrome infammatoire (CRP: 109 mg/l, VS: 90 mm), avec une hyperéosinophile et un syndrome mononucléosique au frottis sanguin. Le mononucléose infectieuse TEST, et les sérologies virales négatives. Les hémocultures étaient positives au Streptocoque oral (S.mitis), L’ECBU stérile avec une protéinurie de 24h négative. Une échocardiographie tranthoracique (ETT) trouvait un VG de taille et de fonction systolique normales avec présence d’une comminucation interventriculaire (CIV) périmembraneuse restrictive mesurant 3 mm et une grande végétation mobile de 17 mm greffée sur la berge supérieure de la CIV (Figure 1, Figure 2, Figure 3, Figure 4). La tomodensitométrie (TDM) thoraco-abdominale ne trouvait qu’une hépatosplénomégalie homogène. La TDM cérébrale, la radiographie des sinus (incidence blondeau) ainsi que le fond d’oeil étaient sans anomalies. Le traitement était axé sur l’administration de vancomycine (30 mg/kg/j en IV réparties en deux doses) pendant 4 semaines et de gentamycine (4 mg/kg/j IV en prise unique) pendant 2 semaines. L’évolution était favorable avec disparition de la fièvre, normalisation de la CRP et ce la VS, disparition de l’hyperéosinophilie et du syndrome mononucléosique.

## Discussion

L’endocardite infectieuse est une pathologie rare avec une incidence annuelle d’environ 10 cas/100.000 habitants. Il s’agit d’une pathologie au pronostic péjoratif, malgré les avancées diagnostiques et thérapeutiques, avec une mortalité avoisinant 20% [1]. Elle peut survenir sur cœur sain, mais le plus souvent il s’agit d’un cœur pathologique [2]; et quoique les EI sur valvulopathies rhumatismales occupent depuis toujours la première place dans les pays en voie de développement [3], le profil des patients atteint d’EI connaît un changement notable dans les pays développés, les EI sur valvulopathies rhumatismales ou dégénératives devenant de moins en moins fréquentes au profit des EI sur cardiopathie congénitale (CC) [4]. Cardiopathies congénitales « corrigées », non opérées ou ayant eu une palliation sont concernées par la greffe bactérienne. La réparation chirurgicale est supposée diminuer voire annuler le risque ; cela n’est vrai que si aucune lésion résiduelle ne persiste. La réparation des cardiopathies complexes comporte la mise en place de prothèses valvulaires, de tubes prothétiques qui constituent des cibles à risque infectieux, ou bien laissent persister des lésions valvulaires ou autres shunts à risque certes plus faible, mais non nul [5]. La communication interventriculaire (CIV) est la seconde cardiopathie congénitale en matière de fréquence après la bicuspidie aortique, mais en est la première en termes d’EI. Différentes études et registres ont trouvé que parmi les EI sur cardiopathies congénitales, la CIV vient en chef de fil; les patients qui en sont porteurs ont six fois plus de risque d’avoir une EI que population générale [4, 6, 7]. La présentation clinique des EI est très variable, La fièvre est pratiquement toujours présente et représente le mode de révélation principal. Un tableau d’insuffisance cardiaque parfois sévère peut être révélateur ou compliquer l’évolution. Environ un tiers des patients présenteront un ou plusieurs emboles, qu’il faut rechercher par un bilan d’extension exhaustif [8].

L’échocardiographie, qu’elle soit transthoracique (ETT) ou transœsophagienne (ETO), est un examen capital dans la prise en charge et le suivi de toute EI. Elle doit être réalisée aussitôt qu’une EI est suspectée. L’ETO est recommandée si le patient est connu porteur d’une prothèse valvulaire ou d’un matériel intracardiaque, si l’ETT est négative avec une forte suspicion clinique d’EI surtout si l’ETT est sous optimale, enfin elle devrait être faite devant toute EI pour éliminer une complication locale [2]. Cependant, le diagnostic échographique peut être mis en échec dans les cardiopathies complexes comportant des anomalies valvulaires et shunts multiples: près de 65% des endocardites infectieuses sur cardiopathies complexes n’ont pas de lésions échocardiographiques évidentes. En effet, les lésions infectieuses sont parfois impossibles à distinguer des anomalies préexistantes ou sont invisibles si localisées sur des shunts palliatifs systémicopulmonaires [5]. Généralement, ces difficultés ne se posent pas dans les EI sur CIV pour lesquelles l’échocardiographiste devra en plus préciser la localisation, la sévérité, la taille et le nombre des CIV mais également évaluer la surcharge volumique du VG et les pressions artérielles pulmonaires [6].

Le dernier point dont on voudrait discuter est la prévention de l’EI en cas de CIV. En effet d’après les dernières recommandations européennes qui sont parues en 2015, l’antibioprophylaxie n’est recommandé que pour les cardiopathies à haut risque- la CIV n’en faisant pas partie - en cas de procédures dentaires à haut risque. the National Institute for Health and Care Excellence (NICE 2008) va encore plus loin et ne recommande guère d’antibioprophylaxie quelque soit la prédisposition cardiaque et la procédure. les sociétés savantes avancent les raisons suivantes pour expliquer les restrictions portées à l’antibioprophylaxie : les bactériémies causées par les activités quotidiennes tel que le brossage des dents durent nettement plus longtemps (5.370 minutes/mois) que celles dues à une extraction dentaire (30 min), les études cas-témoin n’ont pas montré d’association entre les procédures dentaires dites invasives et la survenue d’EI, l’efficacité de l’antibioprophylaxie sur la bactériémie et la survenue d’EI n’a été prouvé que chez la animaux alors que ceci reste une question controversée chez l’Homme, enfin l’administration d’antibiothérapie expose au risque d’anaphylaxie et d’émergence de souches résistantes [1, 2]. Mais on pourrait se demander si ces recommandations peuvent-ils réellement être extrapolées dans les pays en voies de développement? Avec des populations différentes? Le manque d’asepsie périprocédurale dans certaines régions? Etc. ‘’The Adult Expert Review Group for the hospital level’’ appartenant au National Essential Drug List Committee en afrique du sud recommande une antibioprophylaxie oslérienne pour les procédures à haut risque même chez les patients porteurs de valvulopathies acquises [3]. La réponse définitive à l’interêt réel de l’antibioprophylaxie dans la prévention de l’EI nécessite des études randomisées contrôlées qui probablement ne verront jamais le jour vu le nombre très important des sujets à inclure. A notre avis, il est raisonnable d’appliquer les recommandations des sociétés savantes internationales d’une façon pondérée et individualisée en prenant en considérations le contexte socio-économique, l’expertise en médecine dentaire mais également l’environnement microbiologique de chaque région ; l’antibioprophylaxie oslérienne en cas de CIV n’ayant pas de place. Un principe sur lequel toutes les sociétés savantes et groupes d’études se mettent d’accord est la nécessité d’une hygiène cutanée mais surtout bucco-dentaire, souvent négligées par les médecins, chez tous les patients porteurs de cardiopathies à risque avec une consultation stomatologique et dentaire bi-annuelle pour le groupe à haut risque, annuelle pour les autres [1–3].

## Conclusion

Les EI sur CIV sont les plus fréquentes des EI sur cardiopathie congénitale. Leur présentation clinique peut être atypique d’où le rôle primordial de l’échocardiographie qui permet, en plus du diagnostic positif, la recherche de complication et le suivi mais également l’étude des CIV. La prévention dans ces cas passe par une hygiène bucco-dentaire et cutanée optimale et non par une antibioprophylaxie. Ainsi, dès que la CIV est découverte, une consultation chez un stomatologue et un dentiste doivent être programmées et le patient devra être éduqué vis-à-vis de l’intérêt de prendre soins de son état bucco-dentaire quitte à donner aux patients porteurs de cardiopathies à risque d’EI, notamment la CIV, des brosses à dents et dentifrices à des intervalles réguliers dans les milieux les plus défavorisés.
